# Inertial data of daily living tasks in bilateral and unilateral vestibulopathy patients and controls

**DOI:** 10.1038/s41597-025-05943-4

**Published:** 2025-10-16

**Authors:** Gautier Grouvel, Stéphane Armand, Sinan Ghavami, Nils Guinand, Angélica Pérez Fornos, Julie Corre

**Affiliations:** 1 Division of Otorhinolaryngology Head and Neck Surgery, Geneva University Hospitals and University of Geneva, Geneva, Switzerland; 2 Kinesiology Laboratory, Geneva University Hospitals and University of Geneva, Geneva, Switzerland; 3 Research Center of Skeletal Muscle and Movement, Geneva University Hospitals and University of Geneva, Geneva, Switzerland; 4 ENT department of Lausanne University Hospital (CHUV), Lausanne, Switzerland

**Keywords:** Outcomes research, Quality of life

## Abstract

The analysis of human movement outside the laboratory could play a key role for assessing balance mechanisms in daily life, where quality of life is often reduced in patients with vestibulopathy. This dataset proposes data from 9 synchronized inertial sensors acquired during the realization of a set of 15 tasks representative of daily life, under the supervision of an operator. Fifty-nine participants: 19 with chronic bilateral vestibulopathy, 20 with chronic unilateral vestibulopathy and 20 healthy subjects were included in this study. The study aimed to quantify movement patterns, and to identify key balance parameters in each group. This dataset provides a valuable resource for validating motion analysis methods and advancing functional monitoring under real-life conditions.

## Background & Summary

Analyzing human movement remains a challenge in the medical field, particularly for identifying and understanding balance mechanisms and potential risks of falling in vulnerable populations^1^. Human movement is complex, and quantifying it does not provide a single, reliable measure of functional status. This is particularly crucial in patients with vestibulopathy, as current assessments fail to capture the complexity of balance dysfunction^2^. Indeed, balance disorders caused by a loss of vestibular function transforms automatically executed tasks to conscious and tiring efforts, potentially increasing the risk of falls^3,4^, and leading to a deterioration in patients’ quality of life^5^. Although recent studies have increasingly focused on motion analysis in this population^6–9^, most rely on costly, time-consuming optoelectronic motion capture systems, limiting their applicability in real-life settings^10^. In recent years, inertial measurement units (IMUs), typically comprising tri-axial accelerometers, gyroscopes, and sometimes magnetometers have been increasingly used to quantify patient movements^10–13^ outside conventional laboratories. These sensors have real potential for providing access to rapid human motion measurement in an environment close to daily life^14,15^. Despite their growing use, publicly available datasets combining IMU data with daily-life task performance remain scarce.

This study addresses that gap by providing a comprehensive dataset acquired from nine IMUs placed on key anatomical segments of participants performing 15 tasks representative of daily activities^14,16,17^. All tasks were conducted in a semi-standardized environment under operator supervision. The dataset includes recordings from 59 participants: 19 with chronic bilateral vestibulopathy, 20 with chronic unilateral vestibulopathy, and 20 healthy controls.

The primary aim was to quantify movement patterns during task execution to identify key features and tasks that distinguish between participant groups. This work contributes to broader efforts to improve our understanding of functional impairments in everyday life and to enable objective, real-world monitoring of patients.

This dataset^18^ can thus be used independently or in combination with other datasets to validate methods and algorithms for analyzing participants’ movements during the performance of daily life tasks under supervision.

## Methods

### Participants

Nineteen patients (11 females, mean ± sd, age: 60.2 ± 11.6 years, height: 169.1 ± 8.6 cm, weight: 71.5 ± 13.7 kg, BMI: 24.9 ± 3.5 kg/m^2^) with chronic bilateral vestibulopathy (BV), 20 patients (10 females, 9 affected on the left side, mean ± sd, age: 59.5 ± 5.5 years, height: 173.4 ± 10.0 cm, weight: 74.5 ± 13.5 kg, BMI: 24.7 ± 3.4 kg/m^2^) with chronic unilateral vestibulopathy (UV), and 20 healthy subjects (HS) (10 females, mean ± sd, age: 57.9 ± 5.3 years, height: 172.1 ± 8.4 cm, weight: 72.1 ± 13.5 kg, BMI: 24.3 ± 4.1 kg/m^2^) took part of this study. Participant details are available in an additional file in the dataset^18^. BV patients were recruited according to the guidelines of the Classification Committee of the Bárány Society^19^: unsteadiness when walking or standing, oscillopsia and/or worsening of imbalance in darkness/uneven ground, no symptoms while sitting or lying down, bilaterally reduced or absent vestibulo-ocular reflex documented by a caloric test, video-head impulse test (vHIT), or torsion swing test, and finally not better accounted for by another disease. Regarding the UV patients, they had to have a deficit for at least three months and to meet clinical vHIT requirements, with gain values below 0.6 for the lateral semicircular canals of the affected ear, and to have a normal vestibular function in the other ear. Finally, all HS met a criterion of normal vHIT gain values for all semicircular canals (vHIT gain values above 0.8). All study participants were over 18 years of age and provided their written informed consent. The study was designed and conducted in accordance with the guidelines of the Declaration of Helsinki and was approved by the Cantonal Commission for Research Ethics of Geneva (BASEC-ID: 2024-02394).

### Records

**Fig. 1:**
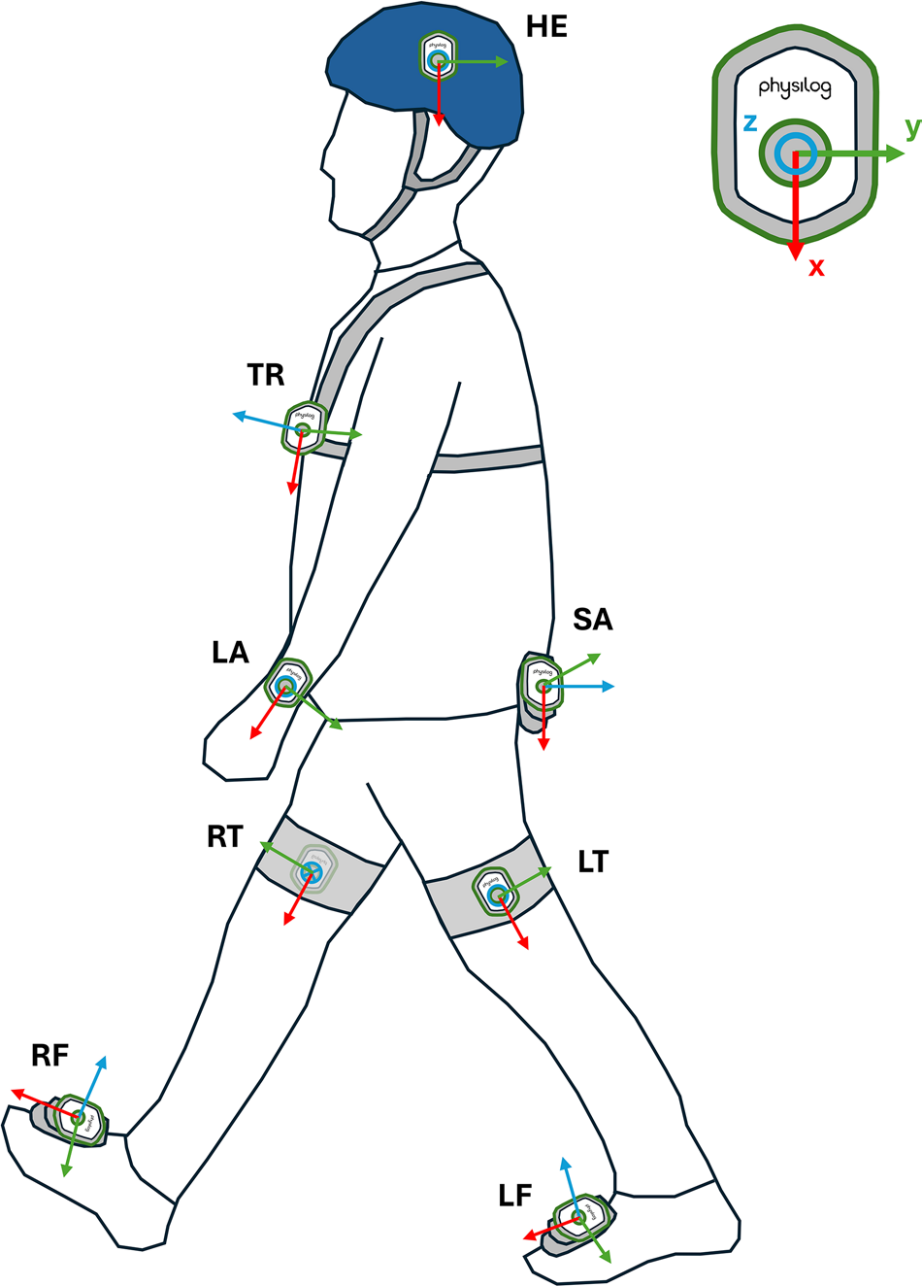
Position of inertial measurement units (IMUs) and definition of the coordinate systems of each sensor used during the measurement. HE: Head; TR: Trunk; SA: Sacrum/Pelvis; LA: Left arm; RA: Right arm (not visible on the figure); LT: Left thigh; RT: Right thigh; LF: Left foot; RF: Right foot.

Nine IMUs (Physilog6S, MindMaze, Lausanne, Switzerland) sampled at 128 Hz were attached to the participants’ head, trunk, pelvis (sacrum), wrists, thighs, and feet (Table 1 & Fig. 1). They recorded 3D linear accelerations with a range of ±16 g, and 3D angular velocities with a range of ±2000 °/s. A short description of IMUs data is presented in Table 2. The IMUs were switched on a few minutes before the start of the recordings, before the participants were equipped. Synchronization between the IMUs was visually checked by synchronous flashing between the LEDs of the 9 IMUs. One IMU configured as “master” received the radio information for synchronization with the 8 other IMUs configured as “slaves”. The “master” IMU was always switched on last, to ensure the same routine for all measurements and approximately the same switch-on delay. After recording, the IMUs were switched off under the same conditions as for switch-on.

**Table 1:** IMU location description and fixation on the participants.

IMU ID	IMU location	Location description	Fixation
HE	Head	Left side of the head	On a climbing helmet using a Velcro fastener
TR	Trunk	On the torso, if possible, under the chest	With a GoPro® harness
SA	Sacrum	On the pelvis, at the level of the sacrum, between the two posterior iliac spines	With a clip attached to the participant’s belt or directly to her/his pants
LA	Left Arm	Posterior left forearm, above the wrist	With a watchband
RA	Right Arm	Posterior right forearm above the wrist	With a watchband
LT	Left Thigh	In the middle of the left thigh on the outer side	With a clip attached to an elastic band fastened to the patient’s thigh
RT	Right Thigh	In the middle of the right thigh on the outer side	With a clip attached to an elastic band fastened to the patient’s thigh
LF	Left Foot	On top of the left foot	With a clip attached to sneaker laces
RF	Right Foot	On top of the right foot	With a clip attached to sneaker laces

**Table 2:** IMU data stored in .csv and .mat files. [BB]: IMU location name; m: number of frames recorded at 128 Hz.

Labels	Format	Dim.	Unit	Description
Timestamps_[BB]	Real	m × 1	s	Digital recording of event time
Acc_x_g_[BB]	Real	m × 1	g	Linear acceleration component X
Acc_y_g_[BB]	Real	m × 1	g	Linear acceleration component Y
Acc_z_g_[BB]	Real	m × 1	g	Linear acceleration component Z
Gyr_x_deg/s_[BB]	Real	m × 1	deg.s^−1^	Angular velocity component X
Gyr_y_deg/s_[BB]	Real	m × 1	deg.s^−1^	Angular velocity component Y
Gyr_z_deg/s_[BB]	Real	m × 1	deg.s^−1^	Angular velocity component Z

### Procedure

For each participant, the entire data collection was performed in a single session which lasted approximately one hour. All sessions were managed by two operators, one in charge of placing the IMUs and performing data recordings, while the other gave instructions to the patient and ensured safety. The following procedure was adopted:

1. *Calibration of the systems:* The calibration of the IMUs was carried out by the manufacturer, and following its recommendations no calibration was required before each measurement
2. *Introduction to the participant:* The operators introduced the environment, the material used and briefly explained the conduct of the session.
3. *Preparation of the participant:* The participant was asked to remove their jacket, and to change their shoes for standardized sport shoes, i.e. identical for each participant. An operator collected the participant’s anthropometric data (height, body mass). Participants were then fitted with IMUs secured by straps or clips (Table 1 & Fig. 1). Other devices were positioned on the participants during the measurements, but are not presented here as the data are not shared.
4. *Measurements:* Participants were asked to perform 15 tasks (Table 3). Before each task, the operator instructed them on how to perform the task with the possibility to skip it if it seemed too complex. The operator who initiated the recording also started the task and ended it when completed by the participant. To start and stop the IMUs at the beginning and end of each task, the operator used a mobile application, developed for the project by the IMU manufacturer.

**Table 3:** List and description of tasks.

Tasks	Description
Bed	The subject lies on his/her back and flat on the bed, remains still for 3 seconds, and then gets up from the bed.
Pants	Standing up, the subject puts a pair of loose pants on, one leg at a time and then takes it off.
Shoes	Without sitting or kneeling, the subject removes his/her shoes one after the other, and then puts them back on.
Sorting	The subject sorts plastic crockery from a storage box onto a shelf as quickly as possible according to color and size.
Heavy load	The subject must carry a heavy load (5 kg) for 10 m, turn around, change hands and return to the starting point. (total: 20 m)
Bus	The subject must press the button to open the door, get on the bus, sit down for a few moments, press the stop button, get up and get off the bus.
Stairs	The subject must climb 8 straight steps and descend 6 spiral steps. If possible, without holding the ramp.
Uneven ground	The subject walks on an unstable cobbled path (15 m).
Tray	The subject must carry two glasses of water on a tray over 12 m without dropping them. The glasses were filled to around 90% of their capacity, with a total weight of approximately 350 g.
Walk	The subject walks in a straight line (12 m) at her/his most comfortable pace.
Stepladder	Subject climbs 5 steps-ladder then comes off.
Wood beam	The subject walks on a 4.5-m wooden beam (15 cm width), turns around and walks back.
Inclined plane	The subject climbs a ramp (25 m - 15°), turns around, and descends it again, the first half of the descent being made with eyes closed.
Picture recognition	Pictures of scenes from daily life are hung on the windows of the room. The subject looks at the pictures (29.7 x 21 cm) while walking (1 round trip: 20 m) and tells the examiner what he/she has seen, giving as much detail as possible. The subject must not stop while walking.
Darkness walk	Subject walks with custom-made darkening glasses (welding glasses) on level ground for 12 m.

All participants also had to complete the Dizziness Handicap Inventory (DHI) self-assessment questionnaire^16^ to evaluate the disabling effects perceived by patients suffering from dizziness. This questionnaire provides insight into patients’ perception of disability. A DHI score between 0 and 30 indicates mild disability, a score between 31 and 60 indicates moderate disability, and a score between 61 and 100 indicates severe disability^20^. The DHI score results are stored in the csv file in the dataset^18^.

### Data processing

After the measurement, IMU data were exported in bin format (manufacturer’s proprietary binary files) by session. A log file was also exported from the phone app containing the start and end events of each task. The IMU data were then loaded into Matlab (R2022b, The MathWorks, USA), synchronized with each other using the manufacturer’s proprietary code, and stored in a Matlab structure for each session. We then sliced the inertial signals using the log file (with start and end timestamps for each task). The sliced data were then exported in .mat format (Matlab file format) and in .csv format. A .mat file containing records for the entire session, i.e. all tasks in a single file, is also present in the dataset. The dataset does not include.bin files because their proprietary format requires a specific program to read the data, which is not compatible with Open Access recommendations. No other data processing was performed on the IMU data.

### Missing data

Some participants did not complete the whole task battery during the session, due to the complexity of the task regarding their symptoms. The list of missing data is presented in Table 4.

**Table 4:** Missing tasks per participant.

Participants	Missing tasks
Participant_04_BV	Pants
Participant_04_BV	Shoes
Participant_04_BV	Wood beam
Participant_09_BV	Pants

## Data Records

All data files are available online on a *Zenodo* database^18^: 10.5281/zenodo.15081742.

The data and metadata in this dataset^18^ comply with MoveD’s guidelines^21^ for movement data publication.

### Data description

The inertial data acquired were recorded for each of the nine IMUs. Time was recorded and stored in the “Timestamps” variable. The inertial data consisted of 3-dimensional linear accelerations and 3-dimensional angular velocities. The data were acquired at a frequency of 128 Hz and stored in variables containing data type (Acc, or Gyr), sensor axis (x, y, or z), unit (g, or deg/s), and sensor location (e.g. HE for Head) – see Table 2 for more details.

Dataset is organized by participant folder,.e.g. Participant_[XX]_[AA] – where XX is the participant number, and AA is the pathological group of the participant (BV: Bilateral Vestibulopathy, UV: Unilateral Vestibulopathy, HS: Healthy Subject). Each folder contains two sub-folders and the following files:

- .csv
  - One .csv file per task containing timestamps and inertial data of the 9 IMUs.
- .mat
  - One .mat file per trial containing timestamps and inertial data of the 9 IMUs.
  - One .mat file containing data from all tasks and sensors.

Csv files are named in our dataset^18^ as IMU_[Task].csv.

Mat files are named in our dataset^18^ as IMU_[Task].mat and IMU_Full_Session.mat.

In .csv files, IMU location information is contained in the name of the column variable, whereas in .mat files, the information is contained in the “Header” variable.

## Technical Validation

### Inertial sensors

No calibration task was recommended by the manufacturer’s documentation, as the IMUs have already been calibrated in the factory. The sensor noises are as follows:

- Accelerometer: average standard deviation <7 mg (milli g-force)
- Gyroscope: average standard deviation <1.3 deg.s^−1^ (degree per second)

## Limitations

The main limitation is the one-time repetition of each task. The low number of participants per group may also be a limitation for using this dataset, especially for machine learning analysis approaches.

In addition, no information was given by the manufacturer of the IMUs, neither for sensor calibration nor for gyroscope zeroing. The gains and offsets were therefore not modified during the measurement.

## Data Availability

The entire dataset is available online in a Zenodo repository. The following URL provides access to the dataset^18^: 10.5281/zenodo.15081742.

## Code availability

No custom code was used, as IMU data were not processed.
